# Genome Sequence of a Lethal Vascular Wilt Fungus, Verticillium nonalfalfae, a Biological Control Used Against the Invasive Ailanthus altissima

**DOI:** 10.1128/MRA.01619-18

**Published:** 2019-01-24

**Authors:** Matt T. Kasson, Lindsay R. Kasson, Kristen L. Wickert, Donald D. Davis, Jason E. Stajich

**Affiliations:** a Division of Plant and Soil Sciences, West Virginia University, Morgantown, West Virginia, USA; b School of Medicine, West Virginia University, Morgantown, West Virginia, USA; c Department of Plant Pathology and Environmental Microbiology, The Pennsylvania State University, University Park, Pennsylvania, USA; d Department of Microbiology and Plant Pathology and Institute for Integrative Genome Biology, University of California, Riverside, California, USA; Vanderbilt University

## Abstract

Verticillium nonalfalfae, a cosmopolitan soil-borne phytopathogen, causes vascular wilt in agricultural crops and perennial woody plants. Select strains of V. nonalfalfae can cause lethal disease in the invasive tree Ailanthus altissima and several have since been utilized as a biological control (biocontrol) against this widespread invader. Here, we report the genome sequence and annotation of V. nonalfalfae strain VnAa140/NRRL 66861.

## Abstract (precis)

Verticillium nonalfalfae, a cosmopolitan soil-borne phytopathogen, causes vascular wilt in agricultural crops and perennial woody plants. Select strains of V. nonalfalfae can cause lethal disease in the invasive tree Ailanthus altissima and several have since been utilized as a biological control (biocontrol) against this widespread invader.

## ANNOUNCEMENT

**FIG 1:**
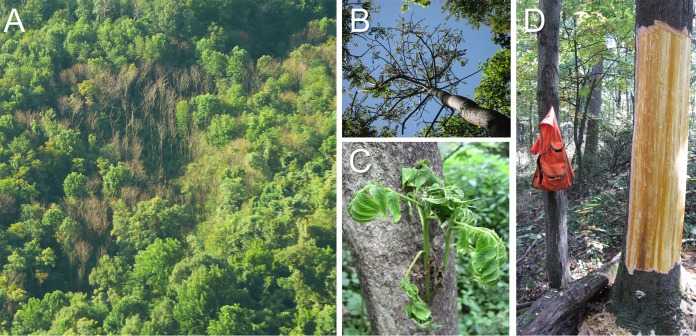
Wilt symptoms in *Ailanthus* trees following artificial inoculation with *Verticillium nonalfalfae* strain VnAa140/NRRL 66861, including, stand dieback and mortality (A); acute wilt and defoliation (B); wilted epicormic sprouts that emerged following dieback of the main stem (C); and conspicuous yellow vascular discoloration and streaking (D).

Since 2005, Verticillium nonalfalfae has been recovered from symptomatic Ailanthus altissima (“tree-of-heaven”) across nine forested locations in the eastern United States. (1–4). These disease epicenters represent natural infections in mixed hardwood stands newly invaded by A. altissima (5). More recently, V. nonalfalfae has been recovered from dying Ailanthus trees in Austria, the first confirmed occurrence on this host outside the United States. (6, 7). This disease is characterized by acute wilting and defoliation followed by epicormic sprouting and mortality, typically within a single growing season (1, 2) (Fig. 1). Several studies using various V. nonalfalfae strains recovered from Ailanthus trees have helped elucidate phylogenetic relationships (2), biocontrol efficacy (2–4, 8), host range (2, 9–11), and transmission (12) in an attempt to understand if and how they differ from strains impacting agronomic crops. Given the ongoing investigations of V. nonalfalfae isolates from hops in Europe (13) coupled with the recent discoveries of V. nonalfalfae in Austria, we anticipate that genome-wide comparisons among Ailanthus trees and hop-origin strains will provide additional insight into host specificity. There also is a renewed interest in utilizing V. nonalfalfae as a biocontrol against Ailanthus altissima in the United States to help combat a newly established invasive insect, Lycorma delicatula, a planthopper that preferentially feeds on this host (14). The generation of genomic resources, including for biocontrol strains, is fundamental to these efforts.

Verticillium nonalfalfae (strain VnAa140/PSU140/NRRL 66861) was isolated from a dying A. altissima in Pennsylvania in 2005 (1, 2). Mycelial fragments and conidia from a 1-week-old culture were transferred to potato dextrose broth and incubated for 2 weeks. Genomic DNA was extracted from the mycelium using a Qiagen DNeasy plant mini kit, and ∼5 µg of input genomic DNA was used to construct sequencing libraries. Illumina HiSeq 2000 sequencing generated 29.3 M paired sequence reads or 3.4 Gb (100× coverage), and 454 sequencing produced 1.3 M singleton reads totaling 523 Mb (16× coverage) at the Pennsylvania State University Huck Institutes Genomics Core. The Illumina sequence reads were assembled with Velvet (v0.7.61) (kmer, 31; -cov_cutoff auto; -ins_length 300; -min_contig_lgth 100; -exp_cov auto) (15). Newbler (v2.3) was used to quality trim the 454 reads by default parameters and generate a hybrid 31.7-Mb assembly (*n* = 630; *N*_50_, 172 kb; max contig, 678 kb; G+C content, 55%) from the Velvet contigs and 454 reads (16). This assembly was cleaned of vector contamination and redundant contigs by Automatic Assembly for the Fungi (AAFTF; v0.2.1) (17) and was further corrected by 5 rounds of polishing with Pilon (v1.22) using the Illumina reads. Genome annotation was performed with funannotate (v1.5.0-760de7c) (18) utilizing available Verticillium dahliae and V. alfalfae transcripts and proteins as evidence (13, 19). The *ab initio* gene predictor GeneMark.hmm ES was self-trained using the default protocol and Augustus parameters trained from alignments of the BUSCO protein set sordariomyceta_odb9 (20–22), and parameters were archived in a public repository (23). The final genome annotation included a total of 9,627 protein-coding genes and 196 tRNAs. AntiSMASH (v4.1.0) predicted 22 putative secondary metabolite clusters (24). This annotation is comparable with that of V. nonalfalfae isolate T2, a lethal xylem-invading hop strain, which had 9,269 protein-coding genes and a total assembly size of 34.2 Mb (13).

### Data availability.

This whole-genome shotgun project has been deposited at DDBJ/ENA/GenBank under the accession number RBVV00000000. The version described in this paper is the first version, RBVV01000000. Sequence reads were deposited under SRA project accession number SRP162963 and BioProject accession number PRJNA493511.
